# Investigating thermal dynamics in cylindrical Li-ion batteries across varied temperatures based on electrochemical principles

**DOI:** 10.1038/s41598-025-16329-2

**Published:** 2025-08-22

**Authors:** Patryck Ferreira, Shu-Xia Tang

**Affiliations:** Department of Mechanical Engineering, Texas Tech University, Lubbock, 79409 USA

**Keywords:** Batteries, Mechanical engineering

## Abstract

Thermal dynamics in cylindrical Li-ion batteries, governed by electrochemical heat generation, are critical to performance and safety in high-power applications such as electric vehicles and grid storage. Building on our previous work, which introduced and validated both single-layer and multi-layer models, this study focuses exclusively on experimentally validating the multi-layer formulation under a broader range of ambient temperatures. The proposed multi-layer model captures temperature evolution across all internal components, including the electrolyte, electrodes, current collectors, and casing, accurately resolving spatial heat accumulation. Experimental validation is conducted across four temperatures (21 $^{\circ }$C, 0 $^{\circ }$C, 40 $^{\circ }$C, and − 10 $^{\circ }$C), demonstrating strong agreement and highlighting the model’s robustness. These results offer actionable insights into internal thermal behavior and may support the design of advanced thermal management strategies, contributing to the development of safer and more efficient Li-ion batteries for next-generation energy storage systems.

## Introduction

Li-ion batteries (LiBs) are essential to modern energy infrastructure, enabling the transition to electrified transportation and large-scale energy storage through their favorable energy density, durability, and low maintenance requirements^1–3^. Yet, their performance and safety are highly sensitive to thermal conditions, particularly in demanding applications such as electric vehicles and stationary storage systems^4,5^. In these contexts, repeated high-current operation and ambient temperature fluctuations can lead to uneven heat distribution, accelerating material degradation and compromising system reliability^6,7^.

During charge and discharge cycles, LiBs generate significant heat, resulting in internal temperature gradients that accelerate degradation and increase the risk of thermal runaway, a dangerous phenomenon driven by uncontrolled temperature increases and exothermic reactions^7–9^. Thermal runaway represents a major safety hazard, potentially leading to fires or explosions, particularly under extreme operational or environmental conditions^4,5^. While advancements in separators and electrolyte additives have reduced some risks, the intricate interplay of thermal, electrochemical, and mechanical processes continues to pose challenges for accurate modeling^10,11^. Furthermore, extreme ambient temperatures intensify these challenges by altering material properties and complicating effective thermal management strategies^12^. Recent advances in experimental techniques have significantly enhanced the ability to characterize thermal behavior in LiBs. For example, a calorimetric method using a cylindrical insulation chamber and temperature sensors was developed to quantify heat generation during cycling^13^. A prismatic-cell calorimeter with controlled boundary conditions was introduced to estimate specific heat and generation rates^14^. A heat flux-based technique was proposed for estimating thermal output in aluminum-air battery systems^15^. Additionally, an in-situ method that compensates for heat loss was developed to accurately characterize heat generation in large-format pouch cells under both high and low temperature-rise conditions^16^.

Accurate thermal modeling of lithium-ion batteries is essential for both safety and performance optimization, particularly under fast-charging and high-power conditions. While finite element approaches using tools like ANSYS^17,18^ and COMSOL^19–21^ enable detailed simulations of coupled electrochemical-thermal behavior, their high computational demands hinder real-time applicability. In contrast, reduced-order models^22,23^ have gained popularity for battery management systems due to their lower computational cost. These include core-surface thermal representations^24,25^ and dynamic field estimation using impedance-based sensing or Kalman filtering techniques^26,27^. To further improve adaptability, recent work has introduced coupled PDE-ODE structures and adaptive observers that account for parameter variations and real-time conditions^28,29^. However, many frameworks still assume uniform heat generation and constant material properties, which can obscure localized hotspots and transient gradients^30^. Detailed interactions involving the electrolyte across components also remain underexplored. Preliminary work has demonstrated that incorporating these effects improves the accuracy of thermal dynamics modeling, particularly when internal temperature gradients are critical for diagnostics and control^31^. The scaling of such models to full battery packs has also been addressed^32^. A recent study introduced both multi-layer and single-layer thermal models to capture temperature dynamics in different battery components, and an observer design was developed but applied only to the single-layer formulation^33^.

Despite these advancements, a critical need remains for models that balance physical accuracy with computational efficiency while resolving detailed internal thermal gradients across battery components. Motivated by these limitations, this study advances the electrochemical-thermal modeling of cylindrical cells by validating a high-resolution multi-layer framework under realistic operating conditions. The key technical contributions of this work are:

- Unlike previous studies that focus on room-temperature conditions^33^, this work extends validation across sub-zero and high-temperature environments, revealing temperature-dependent thermal discrepancies that influence battery performance and safety.
- This validation identifies limitations in temperature-independent modeling assumptions, emphasizing the need for adaptive thermal parameters to improve predictive accuracy, particularly in extreme conditions relevant to electric vehicles, aerospace, and grid-scale storage.

These contributions enhance the practical applicability of high-fidelity thermal models in real-world settings and support future extensions to other cell formats and control-oriented applications.

The article is organized as follows: electrochemical-thermal dynamics presents the cascaded modeling framework, introducing the multi-layer thermal model and electrochemical heat generation via the Single Particle Model (SPM). Experimental setup and model validation describes the test bench, cycling protocols, and model validation under four ambient conditions. A subsection presents simulation results and validation metrics at 21$^{\circ }$C. Performance across ambient temperatures extends the analysis to 0 $^{\circ }$C, 40 $^{\circ }$C, and − 10 $^{\circ }$C, evaluating model robustness and highlighting the effects of using temperature-independent parameters. Broader implications and future directions discusses scalability, applicability to other cell formats, and integration with battery management systems. The conclusion summarizes the main findings and future work. The Methods Section details the mathematical formulations and parameters used in the thermal and electrochemical models.

## Electrochemical-thermal dynamics

The thermal behavior of LiBs is strongly influenced by electrochemical reactions during charge and discharge. In this study, a cascaded modeling framework is employed, in which electrochemical dynamics serve as the source of heat generation that, in turn, drives the temperature response. This hierarchical structure captures the directional coupling between electrochemical activity and thermal dynamics, enabling physically grounded insights into heat distribution and temperature evolution. To characterize these interactions, a high-resolution multi-layer electrochemical-thermal model is presented. The model reflects the spiral-wound geometry of cylindrical cells and resolves temperature distributions in each internal component across all layers. By aligning thermal states with the true physical architecture, the model provides detailed insight into internal temperature gradients that are critical for understanding localized heating and ensuring thermal safety. This approach enables accurate estimation of internal heat accumulation, identification of potential hotspot formation, and evaluation of heat dissipation mechanisms, all of which are essential for optimizing battery safety, performance, and longevity under high-power operating conditions.

### Cylindrical battery structure and thermal modeling

**Figure 1:**
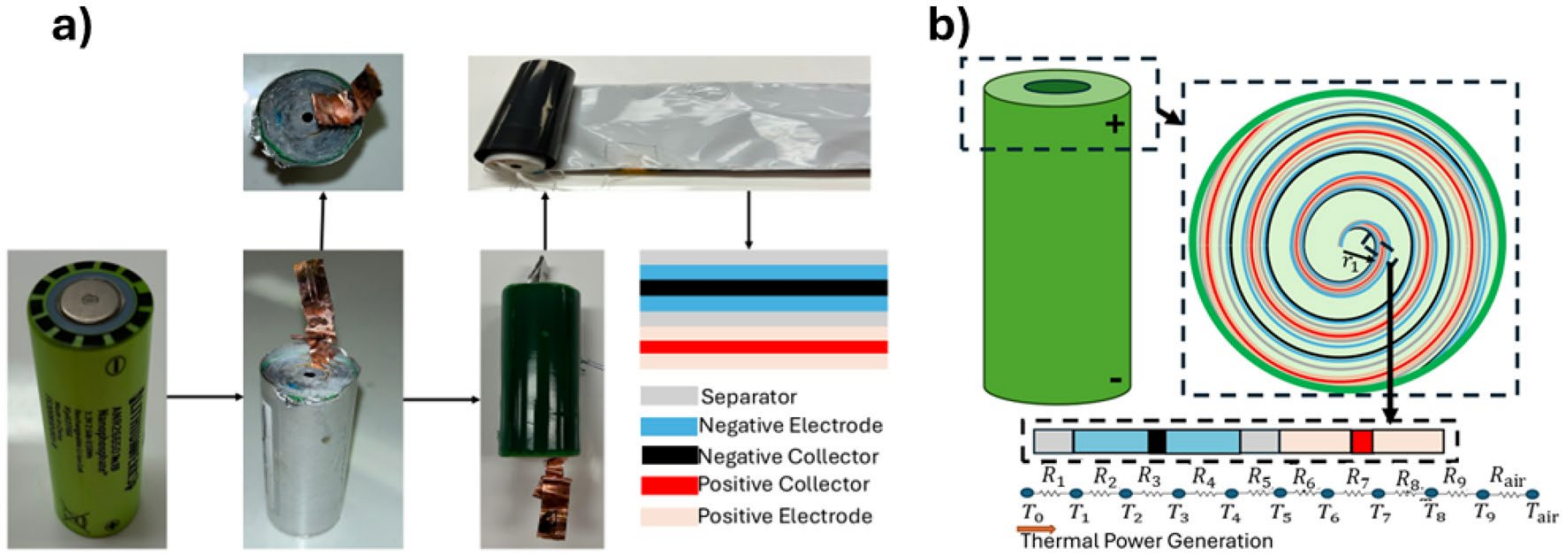
LiB structure^33^. (**a**) Structure of a cylindrical battery; (**b**) Schematic of the spiral-wound structure and the thermal power generation model for each internal component.

As shown in Fig. 1a, the disassembled ANR26650M1B Li-ion cell from Lithium Werks, analyzed in the Distributed Parameter Systems and Control Laboratory at Texas Tech University reveals the spiral-wound configuration typical of cylindrical lithium-ion batteries. The component sequence separator (grey), negative electrode (blue), negative collector (black), positive electrode (pink), and positive collector (red) were confirmed through physical disassembly. These layers repeat throughout the cell, and a total of 38 layers were identified. The layered configuration not only maximizes electrochemical surface area but also strongly influences thermal pathways critical for temperature regulation^33^.

Based on the internal architecture of the battery, a thermal modeling framework is developed to analyze the propagation of temperature between individual components within the spiral wound configuration. As illustrated in Fig. 1b, the model resolves thermal behavior at the level of each material layer, including the electrodes, separators, current collectors, electrolyte, and external casing. The model tracks temperature states for critical regions, including the electrolyte ($T_0$), negative and positive current collectors ($T_3$, $T_7$), electrodes ($T_2$, $T_4$, $T_6$, $T_8$), separators ($T_1$, $T_5$), and the outer casing ($T_9$), along with the corresponding thermal resistances ($R_1$-$R_9$) and the ambient environment ($T_{\text {air}}$, $R_{\text {air}}$). Internal heat generation arises from electrochemical processes and propagates through the battery via conductive and convective pathways. Heat is assumed to originate near the battery core and disperse radially outward, constrained by the thermal resistances of each surrounding layer. This modeling structure enables high-resolution tracking of thermal gradients and dissipation patterns over time, providing valuable insights into heat distribution, which is a critical factor for assessing battery performance and ensuring thermal safety. The thermal architecture and modeling conventions adopted here follow the framework first introduced in our previous work^33^.

### Electrochemical dynamics

**Figure 2:**
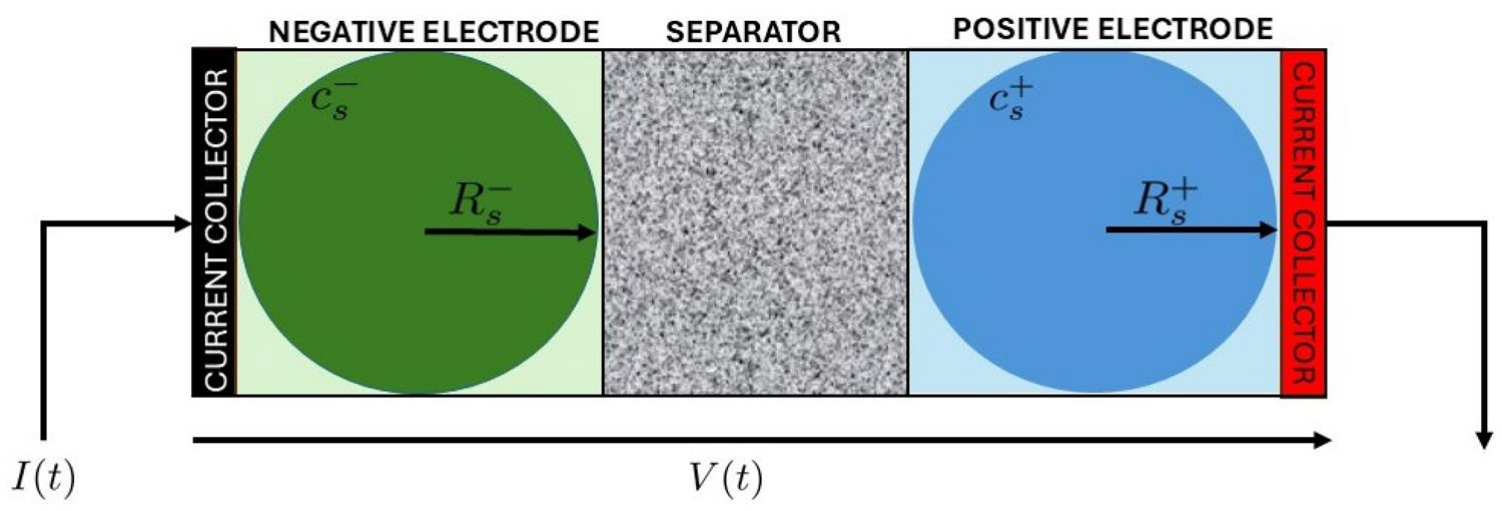
Schematic of the SPM.

To represent the electrochemical processes that generate heat within LiBs, this study adopts the SPM as the underlying electrochemical framework. The SPM describes lithium transport by simplifying each electrode into a single representative spherical particle, capturing lithium diffusion dynamics within the solid phase of the active material. Unlike higher-fidelity models such as the Pseudo-2D (P2D) approach, which resolves both solid-state and electrolyte-phase concentration gradients through complex partial differential equations, the SPM neglects electrolyte dynamics. This assumption significantly reduces computational effort while preserving the core dynamics necessary for capturing the relationship between current input and lithium concentration profiles in the electrodes. The primary advantage of using the SPM lies in its balance between physical interpretability and computational speed, making it particularly suitable for control, estimation, and real-time thermal modeling. A conceptual diagram of the SPM is presented in Fig. 2, which highlights the radial lithium diffusion in the spherical particles and the interaction with external current collectors.

### Integration of electrochemical and thermal dynamics

The formulation of the cascaded electrochemical-thermal model begins with the electrochemical dynamics, which are fully detailed in the Methods Section. The applied current *I*(*t*) is used to drive the SPM, where lithium concentration profiles evolve according to the input (Eqs. (24)–(27)). From these concentration profiles, key intermediate quantities such as the exchange current density (Eq. (23)) and overpotential (Eq. (22)) are derived. The exchange current density and overpotential are then used to compute the terminal voltage *V*(*t*) of the battery (Eq. (20)). The resulting voltage is subsequently used to determine the electrochemical heat generation *S*(*t*) (Eq. (19)), which is then passed into the thermal model. This sequential structure establishes the cascaded interaction whereby electrochemical behavior influences the thermal response. The thermal model applies this heat source to calculate time-dependent temperature evolution across all internal layers using the multi-layer dynamics formulation (Eqs. (10)–(11)).

## Experimental setup and model validation

**Figure 3:**
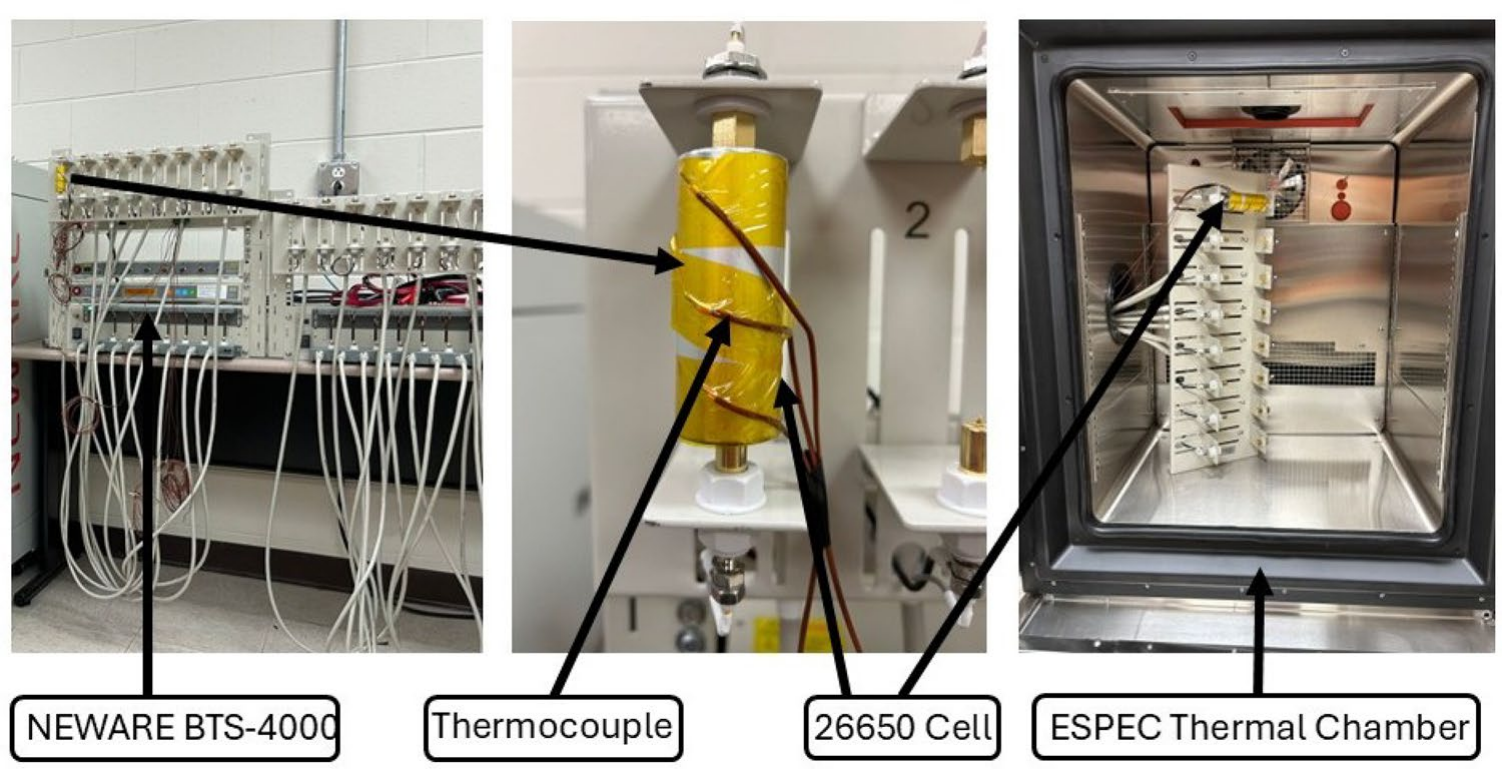
Experimental setup with NEWARE battery tester and ESPEC chamber.

To assess the accuracy of the proposed multi-layer electrochemical-thermal model, experimental validation was conducted in accordance with the verification principles outlined by the American Society of Mechanical Engineers (ASME V&V 10). The testing environment included a BTS-4000 Series 5V12A Battery Tester (NEWARE) and an ESPEC BTU-433 Criterion Benchtop Environmental Chamber. The BTS-4000 provided precise current control with an accuracy of ± 0.05% FS, ensuring consistent and reliable cycling conditions throughout the experiments. Figure 3 shows the experimental setup. Temperature measurements were acquired using Type K thermocouples (NEWARE), each with an accuracy of ± 1$^{\circ }\hbox {C}$.

The experimental tests were performed on a commercially available ANR26650M1B Li-ion cell from Lithium Werks, which is also routinely used in our laboratory. This cell was selected for its high discharge capability, robust construction, and relevance in high-demand applications such as electric vehicles and power tools. A single cell was used consistently across all experiments to maintain controlled conditions and ensure repeatable results, allowing precise validation of the proposed thermal model under identical geometry and usage history. Three sensors were mounted along the external surface using thermally conductive tape to ensure good thermal contact and reliable surface readings. Prior to each test, the environmental chamber was set to the target ambient temperature and allowed to stabilize for one hour to ensure uniform thermal conditions throughout the chamber.

The cycling protocol was programmed using the Neware BTS Battery Testing System, Version 8.0.0, developed by Neware Technology Co., Ltd.^34^, and consisted of four steps: (1) constant current-constant voltage (CCCV) charging at 10 A until reaching a cutoff voltage of 3.45 V, followed by a voltage hold until the current decreased to 0.125 A; (2) a 60 s rest period to allow electrochemical and thermal stabilization; (3) constant current discharging at 10 A down to a cutoff voltage of 2.0 V; and (4) another 60 s rest. This four-step sequence constituted one full charge/discharge cycle and was repeated for the total number of cycles under each temperature condition. The protocol was designed to emulate high-demand usage scenarios while enabling clear observation of heat generation and relaxation behavior during each phase.

To evaluate model performance under varying thermal conditions, experiments were conducted sequentially at four ambient temperatures, with a 24-hour rest period between tests to allow electrochemical stabilization of the cell.

- **21**$^{\circ } {\textbf {C}}$: Served as the baseline representing standard room-temperature operation. A longer test comprising 100 full charge/discharge cycles was conducted to assess long-term thermal behavior under nominal conditions.
- **0**$^{\circ } {\textbf {C}}$: Represents cold conditions typically encountered in winter climates, where reduced electrochemical activity affects efficiency. A shorter test of 10 cycles was used.
- **40**$^{\circ } {\textbf {C}}$: Simulated high-temperature environments that may enhance reaction kinetics but increase the risk of accelerated degradation and thermal instability. This condition was also tested over 10 cycles.
**-10**$^{\circ } {\textbf {C}}$: Reflected extreme cold scenarios such as sub-zero outdoor storage, which significantly increase internal resistance and impair battery performance^35^. This test likewise consisted of 10 cycles.

This order was chosen to first establish a performance baseline at standard conditions and to minimize potential damage from thermal stress before exposing the cell to more aggressive environments. Conducting the 100-cycle test at 21$^{\circ }\hbox {C}$ also enabled robust internal model validation before applying the framework to additional conditions. These diverse conditions were selected to ensure that the model captures thermal behavior across a realistic range of operating environments. This work extends multi-layer model validation to both elevated and sub-zero temperature scenarios. This broader scope enhances the model’s relevance for practical applications where batteries operate under variable thermal loads.

### Simulation setup and model validation

All simulations were performed in MATLAB R2024b, developed by MathWorks, Inc.^36^, on a laptop equipped with an Intel i7-13620H CPU, 32 GB of RAM, and an NVIDIA GeForce RTX 4050 GPU (6 GB). The equilibrium potentials $U^{\pm }(c_{\text {ss}}^{\pm }(t))$, used in calculating the terminal voltage *V*(*t*), are given by the following expressions^33^:

1 $$\begin{aligned} U^+(c_{\text {ss}}^{+}(t))&= 8.3538 - 1.1743 \exp \left( -80.2493 (1 - c_{\text {ss}}^{+}(t))^{1.3198}\right) \nonumber \\ &\quad - 7.8962 \times 10^{-6} \exp \left( 20.2645 (1 - c_{\text {ss}}^{+}(t))^{3.8003}\right) \nonumber \\&\quad + 7.8986 \times 10^{-6} \exp \left( 20.2645 (1 - c_{\text {ss}}^{+}(t))^{3.7995}\right) , \end{aligned}$$

2 $$\begin{aligned} U^-(c_{\text {ss}}^{-}(t))&= 5.3679 + 0.5416 \exp \left( -305.5309 c_{\text {ss}}^{-}(t)\right) + 0.044 \tanh \left( \frac{c_{\text {ss}}^{-}(t) - 0.1958}{0.1088}\right) \nonumber \\&\quad - 0.1978 \tanh \left( \frac{c_{\text {ss}}^{-}(t) - 1.0571}{0.0854}\right) - 0.687 \tanh \left( \frac{c_{\text {ss}}^{-}(t) - 0.0117}{0.0529}\right) \nonumber \\ &\quad - 0.0175 \tanh \left( \frac{c_{\text {ss}}^{-}(t) - 0.5692}{0.0875}\right) , \end{aligned}$$

where $c_{ss}^{\pm }(t) \triangleq c_s^{\pm }(t, R_s^{\pm })$ represents the solid-phase lithium concentration at the particle surface. The governing equations for $c_s^{\pm }(t, r_s)$ are detailed in the Methods Section.

**Figure 4:**
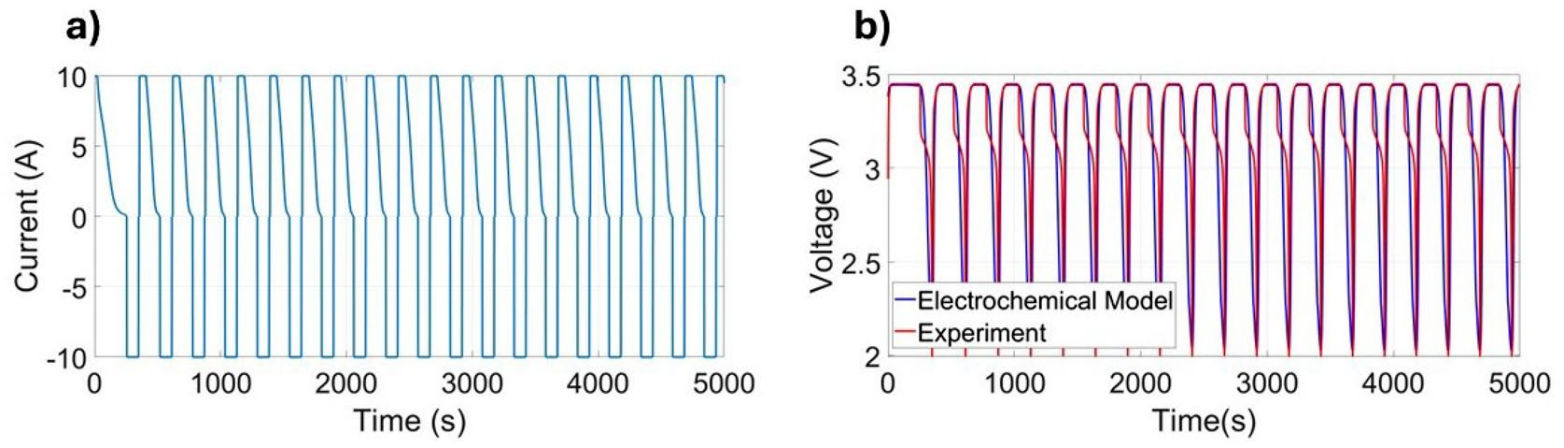
Current and voltage at 21 $^{\circ }$C^33^: (**a**) current profile; (**b**) comparison between the voltage obtained from the SPM and the experimental results.

Figure 4a shows the applied current profile, composed of ± 10 A pulses to reflect high-rate cycling. Figure 4b compares the resulting voltage from the SPM model against experimental data. The curvature observed in the positive current segments in Fig. 4a reflects the transition from constant current to constant voltage during the CCCV charging phase, where the current gradually tapers as the voltage limit is reached. Although each dataset contains the same number of charge/discharge cycles (10), the total duration of the test increases as the ambient temperature decreases. This behavior reflects the intrinsic temperature dependence of the battery’s internal kinetics and transport processes, which become slower at lower temperatures and naturally extend the time required to complete each cycle. The simulated voltage exhibits strong agreement with experimental observations throughout multiple cycling periods, closely matching both the timing and magnitude of transitions. Slight mismatches near voltage extrema likely stem from simplified assumptions that neglect certain secondary electrochemical effects. The model was executed over 100 charge-discharge cycles; however, due to the periodic nature of the profile, only the first 5,000 seconds are shown for clarity. The SPM captures key voltage trends and transitions with reasonable accuracy across multiple cycles. It achieved an RMSE of 0.482 V, which is acceptable for dynamic estimation but may reflect limitations from neglected electrochemical effects.

In the voltage profiles the rest segments appear longer than those in the corresponding current profiles. This visual discrepancy is not an artifact but reflects the inherent dynamic response of the battery. Prior to each rest step, a Constant-Voltage (CV) charging phase is employed, during which the voltage remains clamped at 3.45 V while the current decreases progressively until it reaches the cutoff threshold of 0.125 A. Because the voltage has already plateaued during this phase, it visually blends with the subsequent rest period, even though the current does not reach zero until the CV step completes. As a result, the onset of the voltage rest phase appears earlier than that of the current, although both are technically synchronized.

Beyond this CV effect, the voltage continues to evolve throughout the defined 60 s rest period, while the current remains at or near zero. This behavior is governed by internal electrochemical relaxation processes such as solid-phase lithium diffusion, electrolyte redistribution, and interfacial charge equilibration^37^.

**Figure 5:**
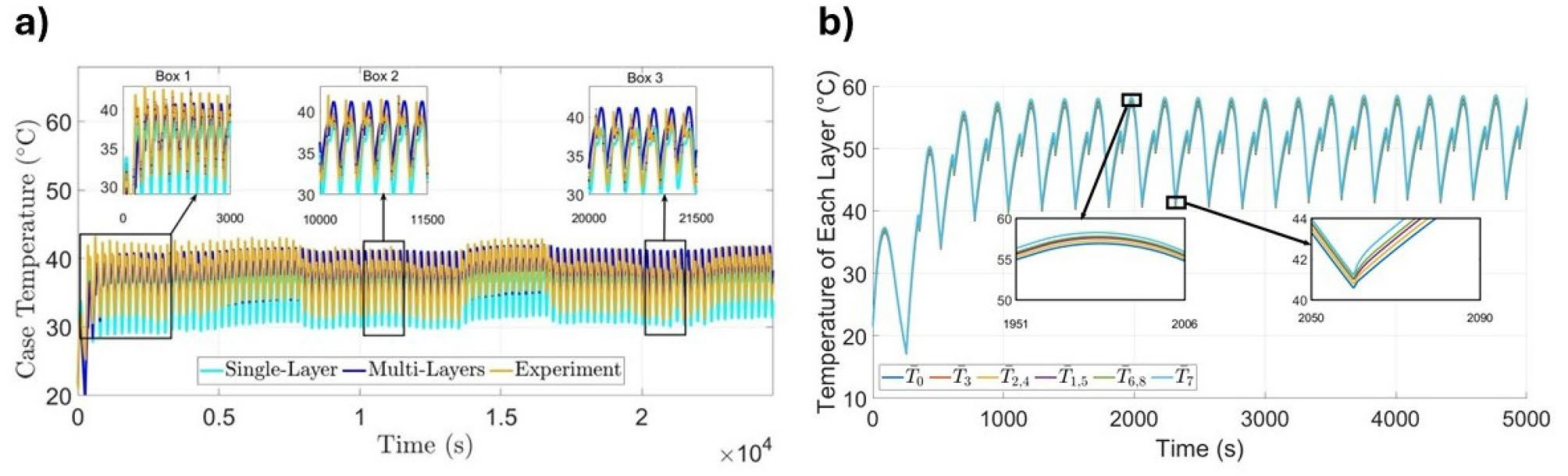
Model validation at 21 $^{\circ }$C^33^: (**a**) surface temperature; (**b**) internal temperature.

The thermal modeling approach is detailed in the Methods Section, where the Multi-layer formulation is derived based on the internal architecture illustrated in Fig. 1b. The multi-layer model resolves the temperature evolution across all 38 internal layers of the 26650-format cell, offering higher spatial fidelity and improved accuracy in capturing internal thermal behavior. Although the single-layer model provides a simpler and more computationally efficient alternative, it lacks the resolution necessary to capture localized heating effects. Before extending the analysis to different ambient temperatures, Fig. 5a demonstrates the importance of internal thermal resolution by comparing the multi-layer model with a simplified single-layer formulation at 21$^{\circ }$C. While both models follow the overall temperature trends, the single-layer model, limited by its single-layer structure, lacks sufficient accuracy in capturing rapid transients and cooling dynamics. In contrast, the multi-layer model reproduces dynamic temperature fluctuations more precisely, especially during rapid cycling events. This result underscores the value of resolving component-level internal temperatures when analyzing heat accumulation and dissipation in cylindrical cells.

While both models track the general temperature profile, the single-layer formulation yielded a higher RMSE of 2.39$^{\circ }$C compared to 1.99$^{\circ }$C for the multi-layer model. This 17% reduction in error confirms that the additional internal structure improves the model’s predictive accuracy. From a modeling perspective, this comparison serves as a verification step, demonstrating that incorporating internal thermal layers offers advantages even when only external data are available for evaluation.

Figure 5b shows the average internal temperature evolution simulated by the multi-layer model for key components such as the electrolyte, separators, electrodes, and current collectors during repeated charge-discharge cycling. The average temperature of each internal component $i \in \overline{1,8}$ is determined by averaging its 38 corresponding temperature states across the battery layers, as defined by: $\bar{T}_i(t) = \frac{1}{38} \sum \limits _{k=1}^{38} T_{38(k-1) + i}(t).$ To simplify the representation, the two separators ($\bar{T}_1,~\bar{T}_5$), two negative electrodes ($\bar{T}_2,~\bar{T}_4$), and two positive electrodes ($\bar{T}_6,~ \bar{T}_8$) are further averaged in pairs: $\bar{T}_{1,5}(t) = \frac{1}{2} \left( \bar{T}_1(t) + \bar{T}_5(t) \right) , \quad \bar{T}_{2,4}(t) = \frac{1}{2} \left( \bar{T}_2(t) + \bar{T}_4(t) \right) , \quad \bar{T}_{6,8}(t) = \frac{1}{2} \left( \bar{T}_6(t) + \bar{T}_8(t) \right) .$ This procedure yields six representative internal temperatures used for visualization and analysis: electrolyte, separator, negative electrode, positive electrode, negative collector, and positive collector^33^. To improve clarity, only the first 5000 seconds of the simulation are displayed, as the thermal response becomes periodic beyond this range. During the initial cycles, the model captures a transient heating phase that stabilizes into a consistent oscillatory profile. Peak internal temperatures approach 58$^{\circ }$C during current application and drop to approximately 40$^{\circ }$C during rest periods, reflecting effective internal heat transfer and energy dissipation throughout the layered cell structure.

Some studies have reported internal thermal gradients using embedded thermocouples. For instance,^38^ demonstrated that internal temperatures in cylindrical cells often exceed external readings during high-current operation. Similarly,^39^ identified radial thermal disparities in jelly-roll configurations, and^40^ observed temperature differences exceeding 10$^{\circ }$C between the center and outer surface of $\hbox {LiFePO}_4$ cells. Due to safety constraints, no internal sensors were used in our experiments. Measurements were limited to surface-mounted thermocouples on the battery case. Direct insertion of sensors into the battery was avoided to reduce the risk of short circuits and thermal runaway. Nonetheless, the multi-layer model at 21$^{\circ }$C predicted internal temperatures up to 15$^{\circ }$C higher than surface values during dynamic cycling, consistent with the behavior reported in the aforementioned studies.

### Performance across ambient temperatures

To evaluate the robustness of the proposed multi-layer thermal model under varying ambient conditions, simulations were performed at 0$^{\circ }\hbox {C}$, 40$^{\circ }\hbox {C}$, and $-10^{\circ }\hbox {C}$. The simulated voltage and temperature profiles were compared against experimental measurements. All thermal parameters and resistances were assumed constant and calibrated at 20$^{\circ }\hbox {C}$. This simplification helps explain the observed cooling delay and minor discrepancies at 0$^{\circ }\hbox {C}$ and 40$^{\circ }\hbox {C}$. In contrast, larger deviations at $-10^{\circ }\hbox {C}$ likely arise from the strong temperature dependence of thermal conductivity, specific heat, and convective coefficients, factors not taken into account in the constant-parameter formulation.

**Figure 6:**
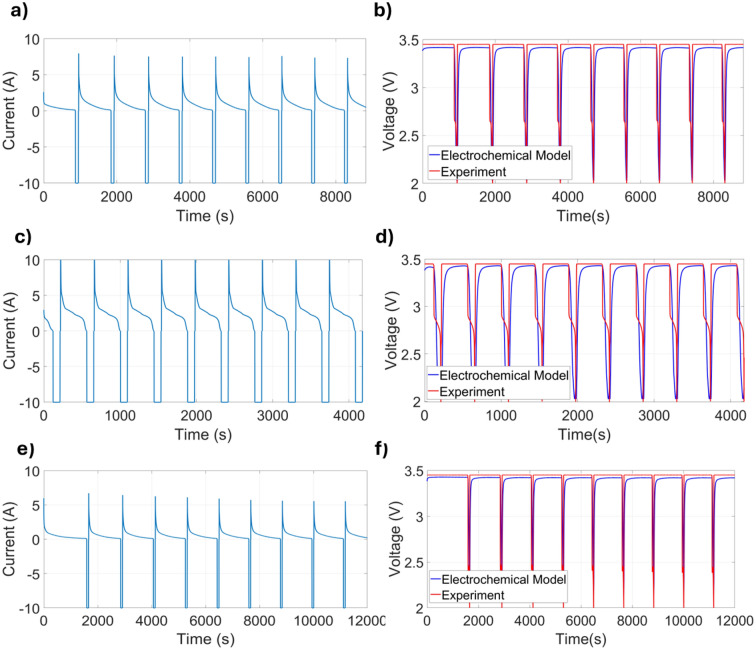
Validation of the electrochemical model under dynamic pulse profiles at different ambient temperatures. Subfigures (**a**,**c**,**e**) show the applied current profiles at 0$^{\circ }\hbox {C}$, 40$^{\circ }\hbox {C}$, and $-10^{\circ }\hbox {C}$, respectively. Subfigures (**b**,**d**,**f**) compare the simulated voltage response from the SPM with experimental data at the corresponding temperatures.

Figures 6a–f present the current and voltage responses at three ambient temperatures: 0$^{\circ }\hbox {C}$ (a) and (b), 40$^{\circ }\hbox {C}$ (c) and (d), and $-10^{\circ }\hbox {C}$ (e) and (f). Figure 6a,c,e show the applied current profiles with alternating ± 10 A. Figure 6b compares the simulated voltage response from the SPM with experimental data at 0$^{\circ }\hbox {C}$, achieving an RMSE of 0.24 V. At 40$^{\circ }\hbox {C}$, Fig. 6d demonstrates a voltage RMSE of 0.46 V, indicating improved voltage prediction under colder conditions. Finally, Fig. 6f shows that voltage simulations at $-10^{\circ }\hbox {C}$ remain accurate, with a low RMSE of 0.18 V. These results confirm that the SPM-based voltage predictions remain reliable across a wide range of temperatures, even when thermal discrepancies are more pronounced.

**Figure 7:**
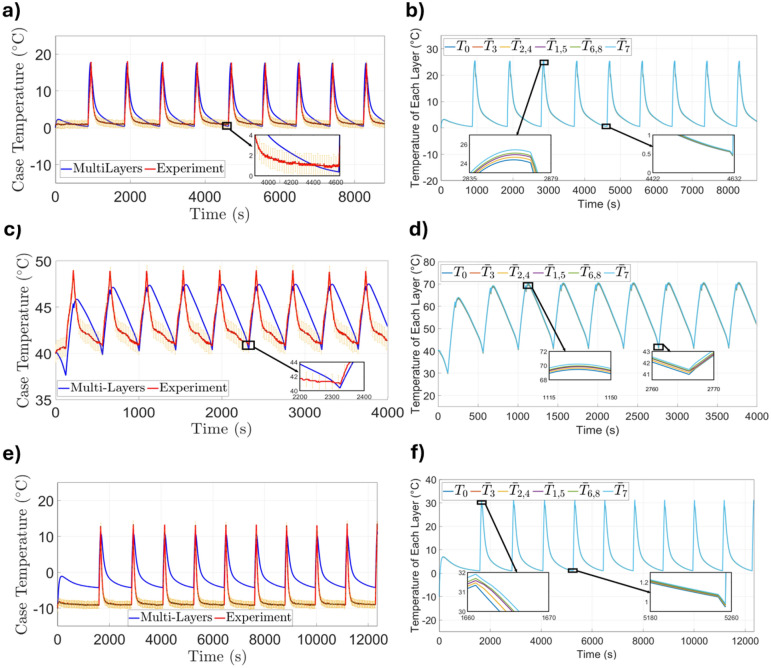
Validation of the multi-layer thermal model at three ambient temperatures: (**a**) 0$^{\circ }\hbox {C}$ and (**b**) 40$^{\circ }\hbox {C}$, (**c**,**d**) 40$^{\circ }\hbox {C}$, and (**e**,**f**) $-10^{\circ }\hbox {C}$. Subfigures (**a**,**c**,**e**) compare simulated and experimental case temperatures, while subfigures (**b**,**d**,**f**) show the internal temperature evolution across multiple layers.

Figure 7 focus on surface and internal temperature behavior across three ambient conditions: 0$^{\circ }\hbox {C}$ (a) and (b), 40$^{\circ }\hbox {C}$ (c) and (d), and $-10^{\circ }\hbox {C}$ (e) and (f). At 0$^{\circ }\hbox {C}$, Fig. 7a shows the case temperature comparison (RMSE = 2.0$^{\circ }\hbox {C}$), and internal temperatures in Fig. 6b display less pronounced lag, consistent with reduced heat generation and smaller thermal gradients. The inset in Fig. 7c reveals a mild delay in cooling, suggesting that temperature-dependent thermal properties may still enhance model fidelity under cold conditions. At 40 $^{\circ }\hbox {C}$, Fig. 7c shows the case temperature comparison with an RMSE of 2.1 $^{\circ }\hbox {C}$, while Fig. 7d displays internal temperature evolution as simulated by the multi-layer model. The model estimates a surface peak temperature of 47.5 $^{\circ }\hbox {C}$, closely aligning with the experimental value of 48.7 $^{\circ }\hbox {C}$. The inset of Fig. 7d highlights that the electrolyte, separator, and electrode layers in the internal layers retain heat longer, revealing a persistent thermal delay. This behavior underscores the importance of accurately capturing internal heat transport under elevated ambient conditions, where cumulative heating can influence safety and performance. Despite the thermal dissipation lag, the model reproduces the overall heating-cooling pattern observed experimentally. Each heating cycle begins and peaks concurrently with the experiment, and the onset of cooling occurs at the same time, though the rate of temperature decrease in the model is slower, resulting in a consistent temporal offset during the cooling phase. At $-10^{\circ }\hbox {C}$, however, the case temperature error increases significantly (RMSE = 6.0$^{\circ }\hbox {C}$, Fig. 7e, while Fig. 7f shows that internal temperatures exhibit pronounced lag and increased peak values, further emphasizing the model’s underestimation of thermal resistance at sub-zero temperatures. The inset in Fig. 7e supports the hypothesis that key thermal properties shift significantly at sub-zero temperatures. These discrepancies highlight the model’s limitations under extreme cold and motivate the incorporation of temperature-dependent thermal properties to improve accuracy for low-temperature applications.

### Impact of ambient temperature on li-ion cell performance

**Figure 8:**
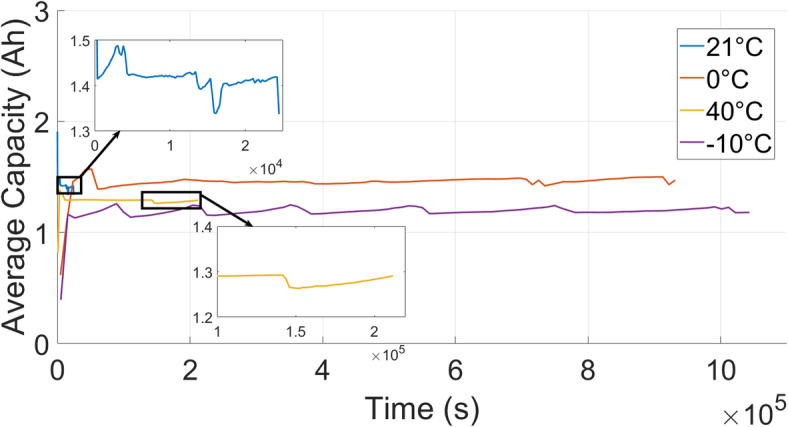
Evolution of discharge capacity over time at four ambient temperatures: $21^\circ$C (100 cycles), $0^\circ$C (100 cycles), $40^\circ$C (100 cycles), and $-10^\circ$C (100 cycles).

Figure 8 illustrates the evolution of average discharge capacity over time under four ambient temperatures. A notable observation is the initially low capacity at $0\,^{\circ }\textrm{C}$ and $40\,^{\circ }\textrm{C}$, followed by a significant increase in subsequent cycles. This phenomenon aligns with previously reported electrochemical behavior, in which $\hbox {LiFePO}_4$ cells exhibited suppressed capacity at low temperatures due to sluggish lithium-ion transport and elevated internal resistance, particularly during early cycles^41^. Such transient behavior may arise from the relaxation of concentration gradients, impedance recovery, and kinetic stabilization. Additionally, the temperature-dependent trends in our data mirror those presented in^41^, confirming that sub-zero ($-10\,^{\circ }\textrm{C}$ ) ambient temperatures accelerate capacity degradation, while moderate temperatures (e.g., $21\,^{\circ }\textrm{C}$ and $40\,^{\circ }\textrm{C}$) maintain relatively stable performance. The capacity jumps observed in our experiments, especially during the early stages, are therefore not necessarily indicative of degradation reversal, but rather reflect transient kinetic limitations that resolve as the cell thermally and electrochemically stabilizes. The time to complete 100 cycles is considerably longer under cold conditions ($0\,^{\circ }\textrm{C}$ and $-10\,^{\circ }\textrm{C}$) compared with $21\,^{\circ }\textrm{C}$ and $40\,^{\circ }\textrm{C}$, reflecting the slower kinetics at low temperature.

### Broader implications and future directions

The multi-layer thermal dynamics model presented in this study enables detailed resolution of internal temperatures in cylindrical LiBs, offering insights into critical phenomena such as hotspot formation and thermal runaway. By capturing thermal dynamics across individual components, the model provides a valuable foundation for developing more effective and reliable thermal management systems. While this study does not directly propose or implement a thermal management strategy, the experimental validation of internal thermal behavior across a wide range of ambient temperatures establishes a foundation for future work in this direction. In particular, the results may support the development of control-oriented approaches and data-driven methods for thermal regulation in cylindrical LiBs.

The demonstrated robustness across different ambient temperatures suggests that the framework is well-suited for real-world operating environments, including both elevated and sub-zero conditions. The use of thermal cameras is planned for future work to visualize temperature distribution across the cell. Moreover, the integration of electrochemical and thermal dynamics supports future investigations into fully coupled multiphysics models, with potential applications in battery control, safety diagnostics, and condition-based maintenance.

While this study focuses on cylindrical cells, the modeling approach is inherently scalable to other battery formats, such as pouch and prismatic cells. Adapting the model to these geometries would primarily require redefining the thermal resistance network based on the corresponding coordinate system and structural layout, as well as adjusting the number and spatial arrangement of internal layers to reflect the specific design of each format. Beyond geometry, the proposed framework also offers a basis for evaluating material properties and structural design strategies aimed at enhancing thermal stability. Collectively, these contributions deepen the understanding of heat transport within LiBs and support the development of safer, more efficient energy storage systems.

## Conclusion

This study presented an electrochemical-thermal model for cylindrical lithium-ion batteries, integrating a detailed multi-layer thermal framework with electrochemical dynamics. The model accurately captures temperature evolution across internal components, providing valuable insights into heat accumulation, thermal gradients, and the effects of ambient temperature variation.

Validation against experimental data under ambient conditions ranging from 0$^{\circ }\hbox {C}$ to 40$^{\circ }\hbox {C}$ showed strong overall agreement, particularly at 21$^{\circ }\hbox {C}$, where model parameters were calibrated. At 0$^{\circ }\hbox {C}$ and 40$^{\circ }\hbox {C}$, a slight cooling delay was observed, attributed to the use of temperature-independent parameters. Nonetheless, the model successfully reproduced the experimental temperature profiles across all tested conditions, demonstrating its robustness and applicability for thermal diagnostics and system design.

At $-10^{\circ }\hbox {C}$, the model exhibited more significant deviations, likely due to the assumption of constant thermal properties. This result highlights the importance of incorporating temperature-dependent parameters to improve predictive accuracy under extreme cold conditions. Despite this limitation, the framework remains physically grounded and computationally efficient, enabling in-depth thermal analysis for high-demand applications.

## Methods

This section outlines the thermal and electrochemical models that constitute the proposed cascaded framework. The derivation begins with the multi-layer thermal model, which captures the temperature dynamics of each internal component within the cylindrical battery cell. This model is constructed based on the real spiral wound architecture of 26650-format lithium-ion batteries and resolves detailed heat transfer across all layers.

Following the thermal formulation, the electrochemical dynamics are described using the SPM, which governs lithium-ion concentration and cell voltage behavior. These electrochemical states are used to compute the internal heat generation, which serves as the input to the thermal model. Together, these components form a hierarchical framework that connects internal electrochemical activity to the temporal evolution of temperature across the battery structure.

The full modeling structure builds upon the cascaded electrochemical-thermal framework previously introduced in^33^, which includes a detailed derivation of an observer-based estimation method. The present study, however, emphasizes the physical interpretation and experimental validation of the model across varied ambient temperatures.

### Thermal model

Building upon our previously introduced framework, the thermal model is developed based on the multi-layer structure of cylindrical lithium-ion batteries, where each internal component is individually modeled to resolve its thermal behavior^33^. For $i \in \overline{0,9} = \{0,1,2,3,4,5,6,7,8,9\}$, the energy balance is expressed as:

3 $$\begin{aligned} \dot{E}_{\text {st},i}(t,r,\theta ,z)&= \dot{E}_{\text {in},i}(t,r,\theta ,z) - \dot{E}_{\text {out},i}(t,r,\theta ,z) + \dot{E}_{\text {g},i}(t,r,\theta ,z), \end{aligned}$$

where $(r, \theta , z) \in \mathbb {R}^3$ are cylindrical coordinates. Here, $\dot{E}_{\text {st},i}$ denotes the rate of energy stored, $\dot{E}_{\text {g},i}$ is the rate of internal heat generation, and $\dot{E}_{\text {in},i}$, $\dot{E}_{\text {out},i}$ are the rates of thermal energy entering and leaving the component, respectively. Due to the layered, spiral-wound configuration, thermal conduction in the axial direction is more efficient than in the radial direction. It is assumed that the battery ends are adiabatic, with minimal axial heat exchange^42^, so the net heat transfer across these surfaces is considered negligible. As a result, Eq. (3) simplifies to:

4 $$\begin{aligned} \dot{E}_{\text {st},i}(t) = \dot{E}_{\text {g},i}(t). \end{aligned}$$

The rate of change of temperature in each layer is given by:

5 $$\begin{aligned} \dot{T}_i(t) = \frac{Q_i(t)}{\rho _i c_{\text {p},i}}, \end{aligned}$$

for $i \in \overline{0,9}$. Here $\rho _i$ and $c_{\text {p},i}$ denote the density and specific heat capacity of component *i*, and $Q_i(t)$ is the net heat input:

6 $$\begin{aligned} Q_i(t) = \frac{S(t)}{\sum \limits _{\begin{array}{c} j=0 \\ j \ne i \end{array}}^{8} v_j}+ \frac{q_{\text {cond},i}(t)}{v_i} + \frac{q_{\text {conv},i}(t)}{v_i}, \end{aligned}$$

with $v_i$ representing the component volume. Here, *S*(*t*) is the electrochemical heat generation, $q_{\text {cond},i}$ is the conductive heat flow between adjacent layers, and $q_{\text {conv},i}$ accounts for convective exchange with the electrolyte. The volume $v_j$ excludes the case.

Conduction between layers follows Fourier’s law:

7 $$\begin{aligned} q_{\text {cond},i}(t) = \frac{\Delta T_i(t)}{R_i}, \end{aligned}$$

where $\Delta T_i(t)$ is the temperature difference across layer interfaces and $R_i$ is the thermal resistance:

8 $$\begin{aligned} R_i = \frac{1}{2 \pi k_i L_i} \ln \left( \frac{r_2}{r_1}\right) , \end{aligned}$$

with $k_i$ denoting thermal conductivity, $L_i$ the axial length, and $r_1$, $r_2$ the inner and outer radii of the layer. Due to the liquid nature of the electrolyte, its thermal conductivity is much lower than that of solid materials, making its role in heat conduction minimal and frequently disregarded in thermal models^43,44^. Consequently, this study assumes $q_{\text {cond},0} = 0$, implying that the thermal resistance $R_0$ within the electrolyte is omitted.

Convective heat exchange between the electrolyte and surrounding components follows Newton’s law of cooling:

9 $$\begin{aligned} q_{\text {conv},i}(t) = hA\Delta T_{i}(t), \end{aligned}$$

where *h* is the convective heat transfer coefficient, *A* is the contact area, and $\Delta T_{i}(t)$ is the temperature difference between the electrolyte and component *i*. No convective transfer is modeled between the electrolyte and the external casing.

#### Multi-layer model

To resolve internal temperature dynamics with high fidelity, a multi-layer thermal model is constructed by explicitly representing each internal component across all layers of the cylindrical battery. For the 26650-format cell examined in this study, the spiral-wound structure comprises 38 repeating layers, each consisting of 8 components. Together with the electrolyte and outer casing, this configuration results in a total of 306 thermal states. Each state evolves according to its own energy balance, yielding a system of coupled ordinary differential equations. This formulation enables fine-grained resolution of heat propagation, accumulation, and dissipation across the entire cell architecture, supporting detailed analysis of internal thermal gradients and potential hotspots. The multi-layer model is defined as:

10 $$\begin{aligned} \dot{\textbf{T}}_\text {multi}(t)&= \mathscr {A}_\text {multi} \textbf{T}_\text {multi}(t) + \mathscr {B}_\text {multi} \textbf{u}(t), \, \end{aligned}$$

11 $$\begin{aligned} \textbf{y}_\text {multi}(t)&= \mathscr {C}_\text {multi} \textbf{T}_\text {multi}(t). \end{aligned}$$

The temperature state vector is denoted by $\textbf{T}_{\text {multi}}(t) \in \mathbb {R}^{306 \times 1}$. The matrices $\mathscr {A}_{\text {multi}}$, $\textbf{T}_{\text {multi}}$, $\mathscr {B}_{\text {multi}}$, and $\mathscr {C}_{\text {multi}}$ are given by:

12 $$\begin{aligned} \mathscr {A}_{\text {multi}}&= \begin{bmatrix} \mathscr {A}_{\text {single}}(1,1:9)&\begin{bmatrix}\begin{bmatrix} \mathscr {A}_{\text {single}}(1,2 :9) \ldots \mathscr {A}_{\text {single}}(1,2 :9) \end{bmatrix}_{37\ \text {times}}0\end{bmatrix}\\ \mathscr {A}_{\text {single}}(2:9, :) & \textbf{0}_{8 \times 296} \\ \textbf{0}_{296 \times 296} & \begin{bmatrix} \mathscr {A}_{\text {single}}(2:9, :) \\ \vdots \\ \mathscr {A}_{\text {single}}(2:9, :) \end{bmatrix}_{37\ \text {times}} \\ \textbf{0}_{1 \times 296} & \mathscr {A}_{\text {single}}(10, :) \end{bmatrix} \in \mathbb {R}^{306 \times 306}, \end{aligned}$$

13 $$\begin{aligned} \mathscr {B}_{\text {multi}}&= \begin{bmatrix} \mathscr {B}_{\text {single}}(1, :) \\ \begin{bmatrix} \mathscr {B}_{\text {single}}(2:9, :) \\ \vdots \\ \mathscr {B}_{\text {single}}(2:9, :) \end{bmatrix}_ \text {304 times} \\ \mathscr {B}_{\text {single}}(10, :) \end{bmatrix} \in \mathbb {R}^{306 \times 2}, \end{aligned}$$

14 $$\begin{aligned} \mathscr {C}_{\text {multi}}&= \begin{bmatrix} \textbf{0}_{1 \times 296}&\mathscr {C}_{\text {single}} \end{bmatrix} \in \mathbb {R}^{1 \times 306}. \end{aligned}$$

The matrices $\mathscr {A}_\text {single}$, $\mathscr {B}_\text {single}$, and $\mathscr {C}_\text {single}$ are constructed by considering a single layer, using (5), (7), and (9) for each component. Substituting these matrices into (12), (13), and (14) allows the model to be expanded to include all layers within the battery. The matrices $\mathscr {A}_\text {single}$, $\mathscr {B}_\text {single}$, and $\mathscr {C}_\text {single}$ are defined as follows:

15 $$\begin{aligned} \mathscr {A}_\text {single}&= \begin{bmatrix} \alpha _1 & -\alpha _2 & -\alpha _3 & -\alpha _3 & -\alpha _3 & -\alpha _3 & -\alpha _3 & -\alpha _3 & -\alpha _3 & 0 \\ -\beta _1 & \beta _2 & 0 & 0 & 0 & 0 & 0 & 0 & 0 & 0 \\ -\gamma _1 & \gamma _2 & \gamma _3 & 0 & 0 & 0 & 0 & 0 & 0 & 0 \\ -\delta _1 & 0 & \delta _2 & \delta _3 & 0 & 0 & 0 & 0 & 0 & 0 \\ -\epsilon _1 & 0 & 0 & \epsilon _2 & \epsilon _3 & 0 & 0 & 0 & 0 & 0 \\ -\zeta _1 & 0 & 0 & 0 & \zeta _2 & \zeta _3 & 0 & 0 & 0 & 0 \\ -\eta _1 & 0 & 0 & 0 & 0 & \eta _2 & \eta _3 & 0 & 0 & 0 \\ -\theta _1 & 0 & 0 & 0 & 0 & 0 & \theta _2 & \theta _3 & 0 & 0 \\ -\iota _1 & 0 & 0 & 0 & 0 & 0 & 0 & \iota _2 & \iota _3 & 0 \\ 0 & 0 & 0 & 0 & 0 & 0 & 0 & 0 & \kappa _1 & \kappa _2 \end{bmatrix} \in \mathbb {R}^{10 \times 10}, \end{aligned}$$

16 $$\begin{aligned} \mathscr {B}_\text {single}&= \begin{bmatrix} 0 & 0 & 0 & 0 & 0 & 0 & 0 & 0 & 0 & \lambda _1 \\ \lambda _2 & \lambda _3 & \lambda _4 & \lambda _5 & \lambda _6 & \lambda _7 & \lambda _8 & \lambda _9 & \lambda _{10} & 0 \end{bmatrix}^{\text {tr}} \in \mathbb {R}^{10 \times 2}, \end{aligned}$$

17 $$\begin{aligned} \mathscr {C}_\text {single}&= \begin{bmatrix} 0&0&0&0&0&0&0&0&0&1 \end{bmatrix} \in \mathbb {R}^{1 \times 10}. \end{aligned}$$

The parameters and their definitions are given by:

$$\begin{aligned} \alpha _1&= \frac{hA}{\Psi _{0}} + \frac{nhA_1}{\Psi _{0}},&\alpha _2&= \frac{hA}{\Psi _{0}},&\alpha _3&= \frac{hA_1}{\Psi _{0}}, \\ \beta _1&= \frac{hA R_1+1}{\Psi _{1} R_{1}},&\beta _2&= \frac{hA R_1-1}{\Psi _{1} R_{1}}, \\ \gamma _1&= \frac{hA_1}{\Psi _{2}},&\gamma _2&= \frac{1}{\Psi _{2} R_{2}},&\gamma _3&= \frac{hA_1 R_{2}-1}{\Psi _{2} R_{2}}, \\ \delta _1&= \frac{hA_1}{\Psi _{3}},&\delta _2&= \frac{1}{\Psi _3 R_{3}},&\delta _3&= \frac{hA_1 R_{3}-1}{\Psi _{3} R_{3}}, \\ \epsilon _1&= \frac{hA_1}{\Psi _{4}},&\epsilon _2&= \frac{1}{\Psi _{4} R_{4}},&\epsilon _3&= \frac{hA_1 R_{4}-1}{\Psi _{4} R_{4}}, \\ \zeta _1&= \frac{hA_1}{\Psi _{5}},&\zeta _2&= \frac{1}{\Psi _{5} R_{5}},&\zeta _3&= \frac{hA_1 R_{5}-1}{\Psi _{5} R_{5}}, \\ \eta _1&= \frac{hA_1}{\Psi _{6}},&\eta _2&= \frac{1}{\Psi _{6} R_{6}},&\eta _3&= \frac{hA_1 R_{6}-1}{\Psi _{6}R_{6}} , \\ \theta _1&= \frac{hA_1}{\Psi _{7}},&\theta _2&= \frac{1}{\Psi _{7} R_{7}},&\theta _3&= \frac{hA_1 R_{7}-1}{\Psi _{7} R_{7}}, \\ \iota _1&= \frac{hA_1}{\Psi _{8}},&\iota _2&= \frac{1}{\Psi _{8} R_{8}},&\iota _3&= \frac{hA_1 R_{8}-1}{\Psi _{8} R_{8}}, \\ \kappa _1&= \frac{1}{\Psi _{\text {9}} R_{\text {9}}},&\kappa _2&= \frac{-R_{\text {air}} - R_9}{\Psi _{\text {9}} R_{\text {9}} R_{\text {air}}},&\lambda _1&= \frac{-1}{\mu _{\text {9}} R_{\text {air}}}, \\ \lambda _2&= \frac{1}{\mu _{\text {0}}},&\lambda _3&= \frac{1}{\mu _{\text {1}}},&\lambda _4&= \frac{1}{\mu _{\text {2}}}, \\ \lambda _5&= \frac{1}{\mu _{{3}}},&\lambda _6&= \frac{1}{\mu _{\text {4}}},&\lambda _7&= \frac{1}{\mu _{\text {5}}}, \\ \lambda _8&= \frac{1}{\mu _{\text {6}}},&\lambda _9&= \frac{1}{\mu _{{7}}},&\lambda _{10}&= \frac{1}{\mu _{\text {8}}}, \end{aligned}$$

where *n* in $\alpha _1$ represents the number of interactions between the electrolyte and each component within a layer, $\Psi _i = \rho _i c_{\text {p}i} v_i$ and $\mu _i = \rho _i c_{\text {p}i} v_j$ for $i \in \overline{0,9}$ and $j \in \overline{0,8}$.

The input vector $\textbf{u}(t)$ is given by:

18 $$\begin{aligned} \textbf{u}(t)&= \begin{bmatrix} T_{\text {air}}(t)&S(t) \end{bmatrix}^\text {tr} \in \mathbb {R}^{2 \times 1}. \end{aligned}$$

Here, $T_{\text {air}}(t)$ represents the air temperature, while *S*(*t*) denotes the electrochemical heat generated by reactions occurring inside the battery.

### Electrochemical heat generation

Electrochemical heat generation, denoted $S(t)$, is computed as:

19 $$\begin{aligned} S(t) = V(t)|I(t)|, \end{aligned}$$

where *V*(*t*) is the terminal voltage simulated by the electrochemical model and *I*(*t*) is the applied current. The absolute value ensures that *S*(*t*) remains non-negative, accounting for thermal energy produced during both charge and discharge phases^45^, Chapter 2, Sect. 2.1. Although heat generation is sometimes approximated as $I(t)^2 R$, this method requires real-time tracking of internal resistance *R*(*t*), which is highly nonlinear and dependent on state of charge and temperature. Instead, the use of *V*(*t*)|*I*(*t*)| implicitly captures resistive losses while remaining computationally efficient.

The terminal voltage *V*(*t*) is modeled as the difference in electric potential between the solid phases of the positive and negative electrodes:

20 $$\begin{aligned} V(t) = \phi _\text {s}^+(t) - \phi _\text {s}^-(t), \end{aligned}$$

where each potential includes contributions from reaction overpotential, open-circuit voltage, and resistive effects:

21 $$\begin{aligned} \phi _\text {s}^{\pm }(t) = \eta ^{\pm }(t) + U^{\pm }(c_\text {ss}^{\pm }(t), T) + F R^{\pm } j^{\pm }(t). \end{aligned}$$

Here, $\eta ^{\pm }(t)$ is the reaction overpotential, $U^{\pm }$ is the equilibrium potential evaluated at the particle surface concentration $c_\text {ss}^{\pm }(t) \triangleq c_\text {s}^{\pm }(t, R_\text {s}^{\pm })$, *F* is Faraday’s constant, and $R^{\pm }$ denotes internal resistance within each electrode.

The overpotential is described by a Butler-Volmer formulation:

22 $$\begin{aligned} \eta ^{\pm }(t) = \frac{R_g T}{\alpha F} \sinh ^{-1}\left( \frac{F}{2 i_0^{\pm }(t)} j^{\pm }(t)\right) , \end{aligned}$$

with $R_g$ being the gas constant, *T* the temperature, $\alpha$ the symmetry factor, and $i_0^{\pm }(t)$ the exchange current density:

23 $$\begin{aligned} i_0^{\pm }(t) = k^\pm \left[ c_\text {ss}^{\pm }(t)\right] ^{\alpha _c} \left[ c_\text {e,0} (c_\text {s}^{\pm ,\max } - c_\text {ss}^{\pm }(t))\right] ^{\alpha _a}, \end{aligned}$$

where $k^\pm$ is the rate constant, $\alpha _c$, $\alpha _a$ are transfer coefficients, and $c_\text {e,0}$ is the reference electrolyte concentration.

The lithium concentration dynamics in the solid phase are governed by the following spherical diffusion equation for each electrode:

24 $$\begin{aligned} \frac{\partial c_\text {s}^{\pm }}{\partial t}(t, r_s)&= \frac{1}{r_\text {s}^2} \frac{\partial }{\partial r_\text {s}} \left( D_s^{\pm }(T(t)) r_\text {s}^2 \frac{\partial c_s^{\pm }}{\partial r_\text {s}}(t, r_\text {s}) \right) , \end{aligned}$$

25 $$\begin{aligned} \frac{\partial c_\text {s}^{\pm }}{\partial r_\text {s}}(t, 0)&= 0, \end{aligned}$$

26 $$\begin{aligned} \frac{\partial c_\text {s}^{\pm }}{\partial r_\text {s}}(t, R_\text {s}^{\pm })&= -\frac{1}{D_s^{\pm }(T(t))} j^{\pm }(t), \end{aligned}$$

27 $$\begin{aligned} c_\text {s}^{\pm }(0, r_\text {s})&= c_\text {s,0}^{\pm }(r_\text {s}), \end{aligned}$$

where $r_\text {s}$ is the radial coordinate within a spherical particle, $D_s^{\pm }(T(t))$ are the solid diffusion coefficients and $j^{\pm }(t)$ are the molar fluxes at the particle surface:

28 $$\begin{aligned} j^+(t) = -\frac{I(t)}{a_\text {s}^+ F L^+}, \qquad j^-(t) = \frac{I(t)}{a_\text {s}^- F L^-}, \end{aligned}$$

with $a_\text {s}^\pm$ as the interfacial surface area, and $L^\pm$ the electrode thickness. This electrochemical formulation enables direct cascading into the thermal model through the computed heat source term *S*(*t*), ensuring that temperature simulations reflect realistic internal reaction behavior under varying current loads.

#### Assumption 1

The solid-phase diffusion coefficients $D_s^{\pm }$ are assumed constant and independent of temperature.

### Thermal and electrochemical parameters

Table 1 presents the thermal and electrochemical parameters used in the simulation, which were obtained from^46^. The multi-layer model employs absolute component volumes to capture the cumulative thermal contributions of the electrolyte, electrodes, separators, collectors, and casing across the entire battery structure. This volumetric fidelity ensures that heat generation and distribution are accurately represented without requiring normalization. Accurately determining thermal conductivity is essential for modeling internal heat transfer in lithium-ion batteries. In this study, constant thermal conductivity values are adopted from established literature^46^. However, recent research has introduced advanced methodologies to account for anisotropic effects. An experimental-numerical framework for quantifying directional thermal conductivities in battery components has been presented, underscoring the value of incorporating anisotropy to improve thermal modeling accuracy^47^.

To streamline the computational modeling of thermal resistances, an average resistance value was calculated based on the mean internal radius of the cylindrical cell. This approximation simplifies the integration of multiple conductive pathways while preserving the overall physical consistency of heat transfer between layers. Given the relatively small variation in radius across successive layers, this simplification offers a practical balance between numerical efficiency and modeling fidelity.

**Table 1:** Parameters used in the simulation.

Category	Parameter	Value/unit
Thermal parameters	Density of electrolyte ($\rho _{0}$)	1130 kg/m$^3$
	Density of negative collector ($\rho _{3}$)	8900 kg/m$^3$
	Density of negative electrode ($\rho _{2,4}$)	2500 kg/m$^3$
	Density of positive collector ($\rho _{7}$)	2700 kg/m$^3$
	Density of positive electrode ($\rho _{6,8}$)	1500 kg/m$^3$
	Density of separator ($\rho _{1,5}$)	1200 kg/m$^3$
	Case diameter ($D_{\text {e}}$)	$25.85 \times 10^{-3}$ m
	Initial layer diameter ($D_{\text {i}}$)	$1.3 \times 10^{-3}$ m
	Specific heat capacity of case ($c_{9}$)	2420 J/(kg K)
	Specific heat capacity of electrolyte ($c_{0}$)	2055.1 J/(kg K)
	Specific heat capacity of negative collector ($c_{3}$)	3440 J/(kg K)
	Specific heat capacity of negative electrode ($c_{2,4}$)	800 J/(kg K)
	Specific heat capacity of positive collector ($c_{7}$)	2420 J/(kg K)
	Specific heat capacity of positive electrode ($c_{6,8}$)	800 J/(kg K)
	Specific heat capacity of separator ($c_{1,5}$)	800 J/(kg K)
	Thermal conductivity of negative collector ($K_{3}$)	398 W/(m K)
	Thermal conductivity of negative electrode ($K_{2,4}$)	1.04 W/(m K)
	Thermal conductivity of positive collector ($K_{6,8}$)	238 W/(m K)
	Thermal conductivity of separator ($K_{1,5}$)	1 W/(m K)
	Thermal resistance ($R_{1}$)	0.0071 K/W
	Thermal resistance ($R_{2}$)	0.0126 K/W
	Thermal resistance ($R_{3}$)	$9.4104 \times 10^{-6}$ K/W
	Thermal resistance ($R_{6}$)	0.0162 K/W
	Thermal resistance ($R_{7}$)	$3.1187 \times 10^{-5}$ K/W
Geometric parameters	Electrolyte volume	$3.0894 \times 10^{-5}$ m$^3$
	Components volume	$3.0894 \times 10^{-5}$ m$^3$
Electrochemical parameters	Faraday constant (*F*)	96,487 C/mol
	Gas constant (*R*)	8.314 J/(mol K)
	Height (*H*)	$65.15 \times 10^{-3}$ m
	Negative electrode diffusion coefficient ($D^-$)	$3.0 \times 10^{-14}$ m$^2$/s
	Positive electrode diffusion coefficient ($D^+$)	$2.5 \times 10^{-16}$ m$^2$/s
	Positive electrode radius ($R_{\text {p}}$)	$3.5 \times 10^{-6}$ m
	Thickness of negative collector ($L_{3}$)	$1 \times 10^{-5}$ m
	Thickness of negative electrode ($L_{2,4}$)	$3.5 \times 10^{-5}$ m
	Thickness of positive collector ($L_{7}$)	$2 \times 10^{-5}$ m
	Thickness of separator ($L_{1,5}$)	$2 \times 10^{-5}$ m

## Data Availability

The datasets used and/or analysed during the current study are available from the corresponding author upon reasonable request.
